# Glucosinolate-Derived Isothiocyanates Inhibit Arabidopsis Growth and the Potency Depends on Their Side Chain Structure

**DOI:** 10.3390/ijms18112372

**Published:** 2017-11-08

**Authors:** János Urbancsok, Atle M. Bones, Ralph Kissen

**Affiliations:** Cell, Molecular Biology and Genomics Group, Department of Biology, Norwegian University of Science and Technology, Høgskoleringen 5, NO-7491 Trondheim, Norway; janos.urbancsok@ntnu.no (J.U.); atle.m.bones@ntnu.no (A.M.B.)

**Keywords:** *Arabidopsis thaliana*, glucosinolates, growth inhibition, isothiocyanates, physiological effects, secondary metabolites

## Abstract

Isothiocyanates (ITCs), the biologically important glucosinolate breakdown products, can present health-promoting effects, play an important role in plant defense and affect plant cellular mechanisms. Here, we evaluated the biological effects of ITCs on *Arabidopsis thaliana* by assessing growth parameters after long-term exposure to low concentrations of aliphatic and aromatic ITCs, ranging from 1 to 1000 µM. Treatment with the aliphatic allylisothiocyanate (allyl-ITC) led to a significant reduction of root length and fresh weight in a dose-dependent manner and affected the formation of lateral roots. To assess the importance of a hormonal crosstalk in the allyl-ITC-mediated growth reduction, the response of auxin and ethylene mutants was investigated, but our results did not allow us to confirm a role for these hormones. Aromatic ITCs generally led to a more severe growth inhibition than the aliphatic allyl-ITC. Interestingly, we observed a correlation between the length of their side chain and the effect these aromatic ITCs caused on *Arabidopsis thaliana*, with the greatest inhibitory effect seen for 2-phenylethyl-ITC. Root growth recovered when seedlings were removed from exposure to ITCs.

## 1. Introduction

Naturally-occurring isothiocyanates (ITCs) can be generated by plants belonging to the Brassicales order when a particular group of nitrogen- and sulfur-containing secondary metabolites called glucosinolates are degraded. This usually happens when plant tissue is disrupted upon mechanical damage or herbivory, bringing together the otherwise spatially separated glucosinolate substrates and β-thioglucosidases called myrosinases which catalyze their hydrolysis into various biologically important phytochemicals [1,2]. The diversity of degradation products is mainly determined by the variable structures of the glucosinolate side chain, which originate from their precursor amino acids. More than 130 different glucosinolates have been identified so far [3]. The outcome of glucosinolate hydrolysis strongly depends on the reaction conditions, taking into consideration the presence or absence of specific proteins and cofactors, and thus hydrolysis products such as nitriles and epithionitriles, can be produced instead of ITCs [4,5,6].

While intact glucosinolates are considered biologically inactive, their degradation products have many documented biological effects. In human and animal cells ITCs affect for example, phase I and phase II enzymes, modulate the cell cycle, and lead to programmed cell death [7,8]. ITCs play also an important role in plant defense against pests and pathogens [9,10]. Glucosinolate-containing plants have also been used in biofumigation to control soil pests and weeds [11], and several ITCs have been shown to inhibit seed germination [12,13]. Applying ITCs directly onto plant leaves leads to phytotoxic effects such as chlorosis, cell death and reduced growth, and can ultimately lead to plant death [14,15,16,17]. At the plant cellular level ITCs have so far been shown to trigger the closure of stomata, to lead to the production of reactive oxygen species (ROS), to lead to glutathione depletion, to affect the cell cycle, to disintegrate microtubules, to inhibit actin-mediated intracellular transport, and to lead to important changes in gene expression profiles in *Arabidopsis thaliana* [17,18,19,20,21,22,23].

Root growth and development is regulated by crosstalk between different plant hormones, where auxin and ethylene are central players in aspects such as primary root growth and the formation of lateral roots [24,25,26].

Auxin has a bimodal effect on *A. thaliana* root length. Exogenously applied auxin promotes or inhibits root growth depending on the concentration [27]. Auxin biosynthesis occurs in the root apex, but the main source of auxin accumulating in the root tip is by polar transport from the shoot, largely via the action of directional transporters such as the auxin efflux carrier proteins PINs, lateral redistribution, and subsequent movement towards the shoot through the external layers. This generates an auxin gradient in the root that is essential for proper root development [25,28].

Lateral root formation starts with the division of quiescent pericycle cells of the parent root that are adjacent to the protoxylem poles, and lateral root formation requires proper auxin biosynthesis, signaling and transport [29]. Higher levels of auxin, such as in auxin overproduction mutants or by exogenous application, lead to more lateral root formation, while auxin deficient mutants are impaired in lateral root formation [25]. Several mutants with a gain of function in the auxin signaling components Aux/IAAs have an altered capacity to form lateral roots, indicating that Aux/IAAs are negative regulators of lateral root formation [24]. Auxin transport also plays a role in lateral root formation, as mutants in the auxin influx carriers AUX1 and LAX3 [30,31] and in some PIN auxin efflux carriers [32,33] have reduced lateral root formation.

Ethylene affects root growth by inhibiting elongation of cells that leave the root meristem, mostly through a stimulation of auxin biosynthesis and transport [34,35], and by modulating the division of cells of the quiescent center [36]. In lateral root formation ethylene acts antagonistically to auxin [37]. Ethylene positively regulates the transport of auxin and blocks changes in the abundance of local auxin transport proteins, which is believed to inhibit local auxin maxima in the protoxylem pericycle of the mature region of the root that are needed to drive lateral root formation [38]. Blocking ethylene responses, such as by the *ethylene triple response 1* (*etr1*) or *ethylene insensitive 2* (*ein2*) mutations, increases lateral root formation [39].

Glucosinolate hydrolysis products like ITCs are mainly produced upon damage of plant tissue, such as under attack by pests and pathogens or mechanical disruption. Most of these products are volatile and released into the gas phase [40]. Glucosinolate levels and profiles change during the plant life cycle, indicating that glucosinolate turnover might also take place in intact tissue [41,42,43]. Plants may therefore be naturally exposed to the effects of their own glucosinolate hydrolysis products in different ways and under different circumstances, but little is known about these effects. Previous studies have shown phytotoxic and growth-inhibiting properties of ITCs when exogenously applied at high (mM to M) concentrations [15,17]. In the present study, several aliphatic and aromatic ITCs were investigated in a µM to mM range for their effects on root growth and biomass production of the model plant *A. thaliana* under standardized in vitro conditions. We observed that concentrations at the 10 to 100 µM level led to reversible root growth inhibition, but that the potency depended on the side chain structure of ITCs. Auxin and ethylene did not seem to play a major role in the ITC-triggered root growth inhibition.

## 2. Results

### 2.1. Allylisothiocyanate (Allyl-ITC) Inhibited Root Growth of Arabidopsis thaliana In Vitro

**Figure 1:**
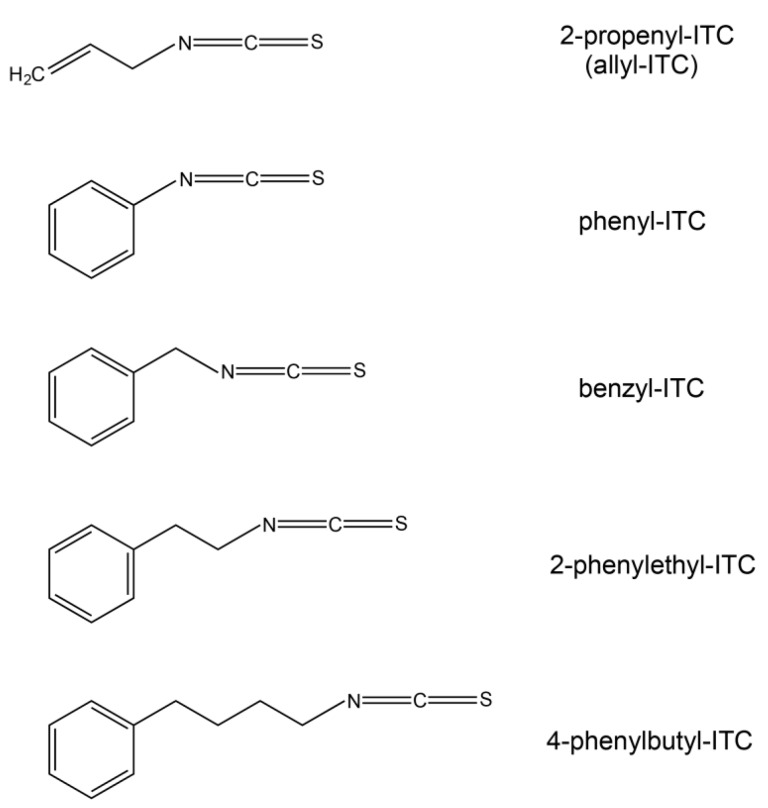
Chemical structures of the isothiocyanates used in this study. ITC: isothiocyanate.

Previous studies indicated that the growth of *A. thaliana* was inhibited when exposed to high concentrations of allyl-ITC (mM to M). In the present study, *A. thaliana* (accession Col-0) was therefore grown on solid in vitro medium supplemented with allyl-ITC (Figure 1) at a range of concentrations exerting no to almost complete root growth inhibition.

**Figure 2:**
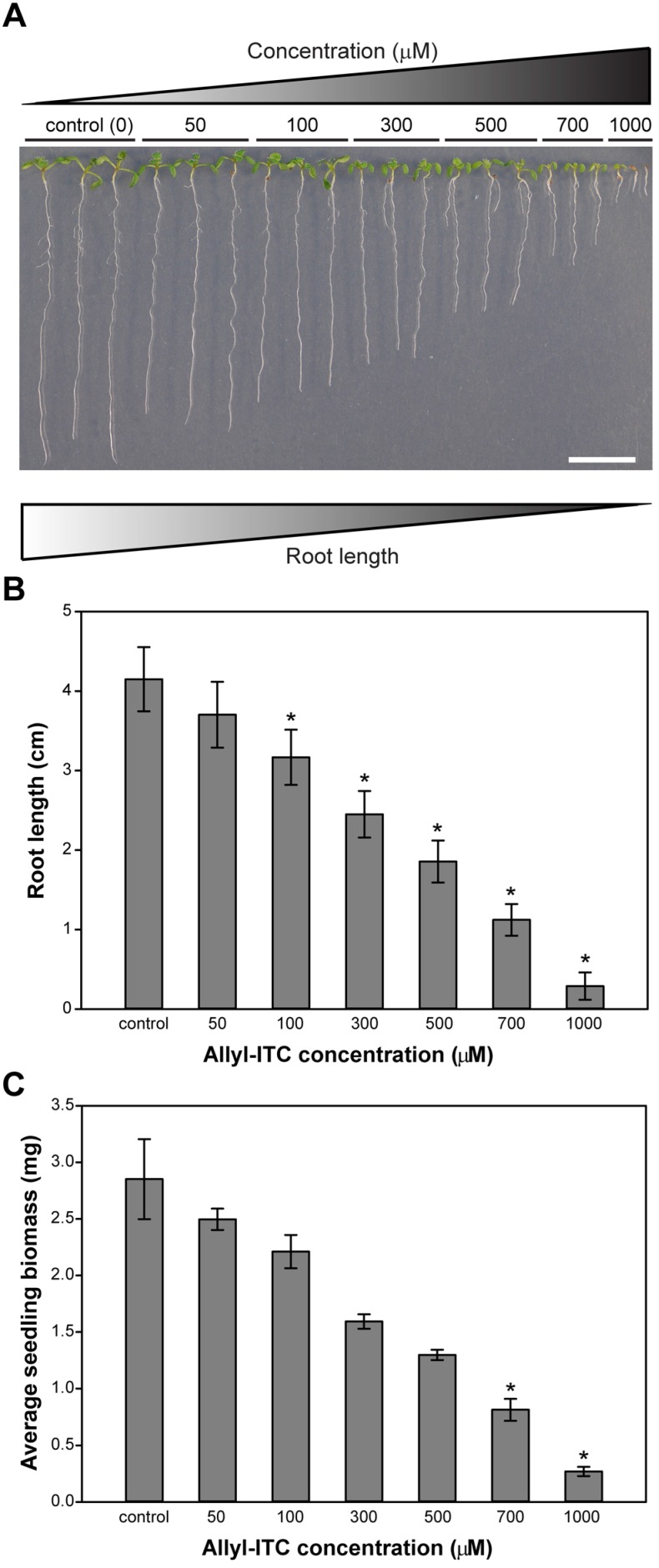
Effect of allylisothiocyanate (allyl-ITC) on the growth of *Arabidopsis thaliana* plants. (**A**) Pictures of representative seedlings grown for 10 days in a vertical position on solid in vitro medium supplemented with different concentrations of allyl-ITC (50 to 1000 µM). Scale bar = 1 cm; (**B**) Average length of the main roots after 10 days on solid in vitro medium supplemented with different concentrations of allyl-ITC (*n* > 48; Kruskal–Wallis One Way Analysis of Variance on Ranks and a post hoc Dunn’s test; * *p* < 0.001; error bars represent SDs); (**C**) Average biomass of seedlings after 10 days on solid in vitro medium supplemented with different concentrations of allyl-ITC (*n* = 4; Kruskal–Wallis One Way Analysis of Variance on Ranks and a post hoc Dunn’s test; * *p* < 0.05; error bars indicate SDs).

This range, from 50 to 1000 µM, showed that allyl-ITC inhibited growth of the main root in a dose-dependent manner (Figure 2A). Root length inhibition ranged from 10% at 50 µM allyl-ITC to an almost complete inhibition (88%) at 1000 µM allyl-ITC (Figure 2B).

### 2.2. Allyl-ITC Reduced the Total Biomass of Arabidopsis thaliana In Vitro

Allyl-ITC showed also a dose-dependent inhibitory effect on the overall plant biomass (Figure 2C). Concentrations of 700 and 1000 µM allyl-ITC reduced the biomass values, respectively, by about 71% and 90% of the control treatment. The lower concentrations of allyl-ITC did not have a significant inhibitory effect on the total plant biomass (Figure 2C).

### 2.3. Allyl-ITC Induced Other Root Morphogenic Changes

**Figure 3:**
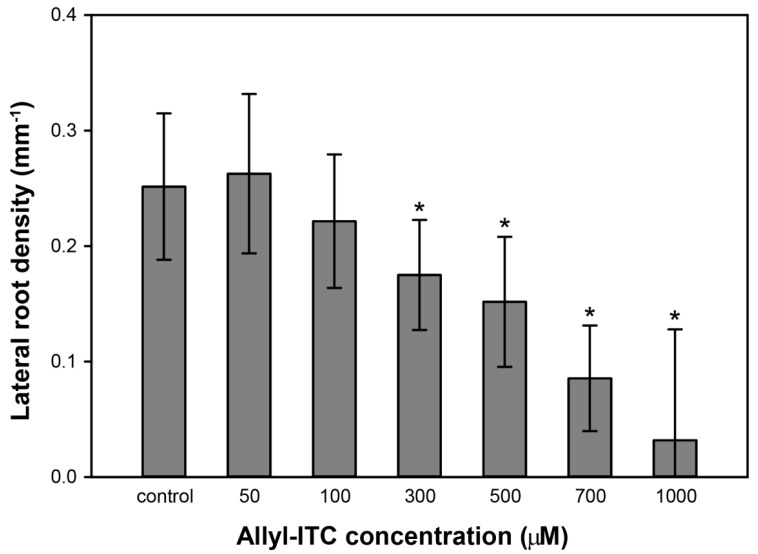
Allyl-ITC effect on lateral root formation. Lateral root density of seedlings grown for 10 days on solid in vitro medium supplemented with different concentrations of allyl-ITC (*n* > 35; Kruskal–Wallis One Way Analysis of Variance on Ranks and a post hoc Dunn’s test; * *p* < 0.001; error bars indicate SDs).

Roots and shoots of 10 day old seedlings exposed to allyl-ITC were also analyzed for other morphogenic changes. When monitoring the number of lateral roots and the region they extended from, we observed that allyl-ITC concentrations of 300, 500, and 700 µM led to a higher number of lateral roots extending from the hypocotyl–root junction, compared to the control treatment. In contrast, the number of lateral roots extending from the main root decreased, leading to a reduction in lateral root density (Figure 3).

### 2.4. A Role for Auxin or Ethylene in Root Growth Inhibition Triggered by Allyl-ITC Could Not Be Established

**Figure 4:**
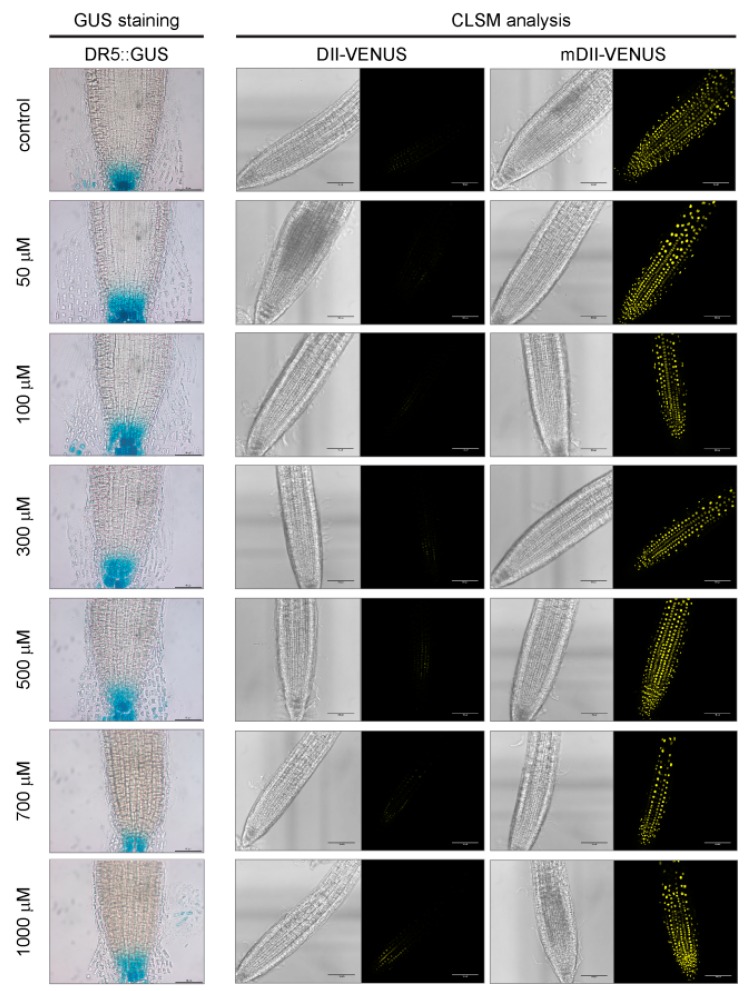
Lack of auxin response to allyl-ITC exposure. Representative pictures of auxin marker lines showing GUS staining of DR5::GUS roots (left panel; scale bar = 50 µm) and YFP fluorescence in roots of DII-VENUS and mDII-VENUS seedlings (right panel; scale bar 100 µm) after a 10 day exposure to the indicated concentrations of allyl-ITC. For DII-VENUS and mDII-VENUS, brightfield images are shown to the left of the corresponding confocal laser scanning microscopy (CLSM) images. The line mDII-VENUS serves as control for putative direct effects of allyl-ITC on VENUS protein levels.

Root development is regulated by many processes, including a crosstalk between auxin and ethylene [37,44], and the expression of auxin- and ethylene-related genes is affected after short time exposure to allyl-ITC [20]. Auxin marker lines were therefore used to assess if also a long-term exposure to allyl-ITC affected the auxin response (Figure 4). Seedlings of the DR5::GUS [45] and DII-VENUS [46] reporter lines responded, as expected, to allyl-ITC by a reduced root elongation similar to Col-0 wild type seedlings. But no changes in GUS staining or YFP fluorescence which would indicate an altered auxin response due to the allyl-ITC treatment, were observed (Figure 4). In addition, none of the auxin response mutant lines that we tested (Table 1) showed a difference to the wild type in the allyl-ITC-induced effect on root length after 10 days of exposure, besides the morphological differences inherent to some of the auxin mutants. Root length inhibition by allyl-ITC of the two ethylene insensitive mutants *etr1-3* (*ethylene response 1-3*) and *ein2-1* (*ethylene insensitive 2-1*) (Table 1) after ten days was not different to that of wild type seedlings either.

### 2.5. Effects on Arabidopsis Growth Depended on ITC Side Chain Structure

**Figure 5:**
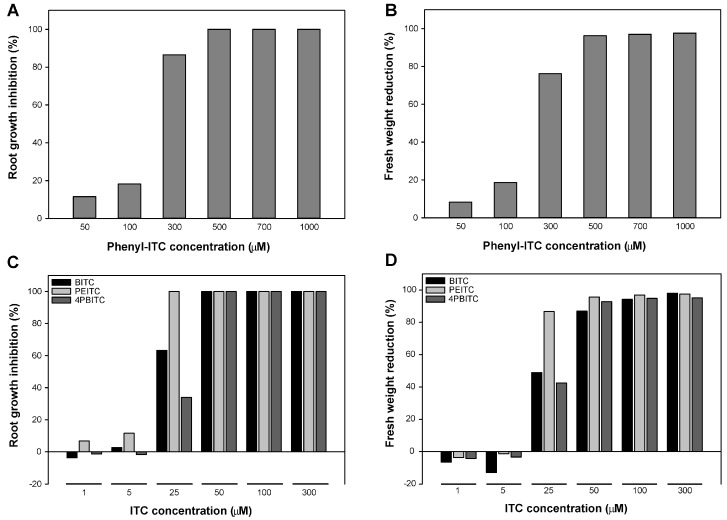

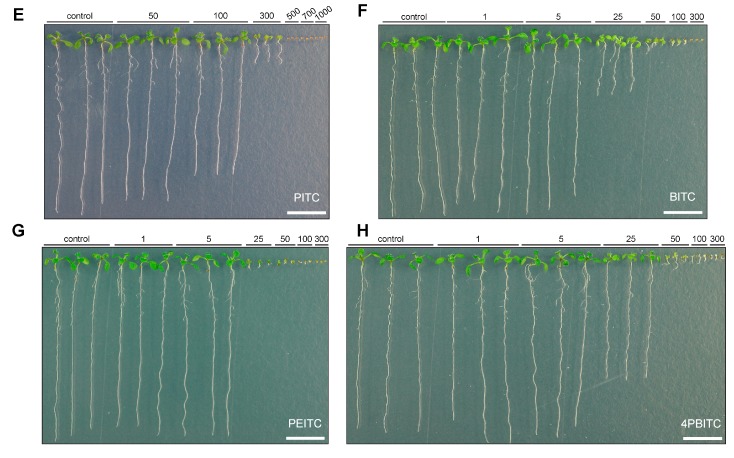
Effect of aromatic ITCs on the growth of *A. thaliana* plants. Inhibition of the main root length (**A**,**C**) and reduction in biomass (**B**,**D**) of seedlings after 10 days on solid in vitro medium supplemented with different concentrations of (**A**,**B**) phenyl-ITC and (**C**,**D**) benzyl-ITC (BITC), 2-phenylethyl-ITC (PEITC) or 4-phenylbutyl-ITC (4PBITC). (**E**–**H**) Pictures of representative seedlings after 10 days on solid in vitro medium supplemented with (**E**) phenyl-ITC (PITC); (**F**) benzyl-ITC (BITC); (**G**) 2-phenylethyl-ITC (PEITC) and (**H**) 4-phenylbutyl-ITC (4PBITC) at the indicated concentrations (µM). Scale bars = 1 cm. Each root growth inhibition percentage was generated using the average root length of at least 48 seedlings growing on a given ITC-supplemented medium divided by the average root length of at least 48 seedlings growing on the control medium (*n* > 48). Each percentage of fresh weight reduction was calculated from the pooled biomass of 15 seedlings per plate (*n* = 4) for the given ITC treatments compared to the control treatment.

To assess if the structure of the side chain had an impact on the growth inhibitory effect of the compound, four ITCs with an aromatic side chain of varying length (Figure 1) were also tested. Similar to the allyl-ITC exposure assay described above, concentrations of each ITC were chosen to cover a range giving no to almost complete root growth inhibition. Phenyl-ITC concentrations of 50 and 100 µM reduced root length by about 11% and 18%, respectively, compared to the control treatment (Figure 5A,E). While 300 µM phenyl-ITC inhibited root growth by more than 85%, concentrations of 500 µM or higher led to a complete inhibition. Benzyl-ITC (Figure 5C,F) and 4-phenylbutyl-ITC (Figure 5C,H) had a clear inhibitory effect on the main root length already at 25 µM. Moreover, benzyl-ITC treatment led to stronger inhibition than 4-phenylbutyl-ITC. On concentrations of benzyl-ITC and 4-phenylbutyl-ITC higher than 25 µM, seeds germinated, but root growth was quickly arrested. At the two lowest concentrations (i.e., 1 and 5 µM) that were tested, no root growth inhibition was observed. 2-phenylethyl-ITC had the strongest effect of all tested ITCs on root growth (Figure 5C,G), with a dramatic reduction of root length occurring when increasing the dose from 5 to 25 µM.

While seed germination was inhibited or slightly delayed at some of the highest ITC concentrations that were tested (Figure 5; Figure S1), the ITC-triggered reduction of primary root length measured after ten days was due to a post-germination inhibition of root growth, and not due to a delayed germination.

As to the effect of aromatic ITCs on plant biomass, phenyl-ITC showed again a clear effect at concentrations of 300 µM and higher, inhibiting biomass by more than 75% (Figure 5B). For the three other aromatic ITCs (benzyl-ITC, 2-phenylethyl-ITC and 4-phenylbutyl-ITC), seedlings hardly developed at this dose (Figure 5F–H), and they showed a significant inhibition of biomass already at a ten times lower concentration (Figure 5D). Similar to root growth, 2-phenylethyl-ITC exerted the highest inhibition, reducing biomass by more than 85% at 25 µM (Figure 5D).

### 2.6. Root Growth Recovered When Seedlings Were Removed from Exposure to ITCs

**Figure 6:**
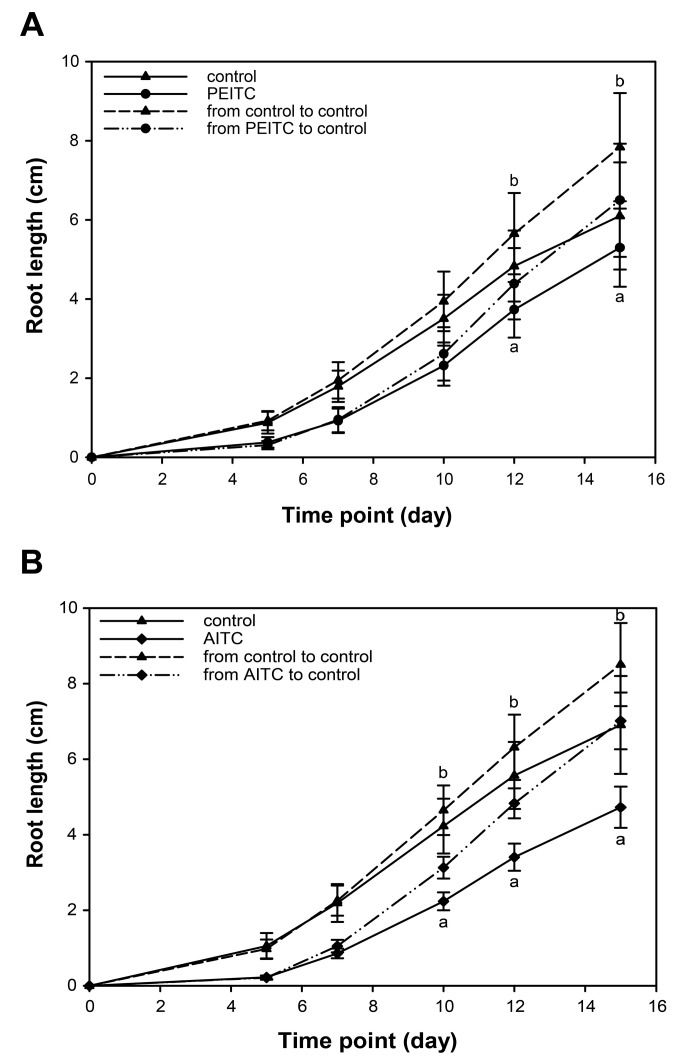
Root growth recovery upon removal of ITC treatment. Root length of seedlings that were grown for five days on solid in vitro control medium, or medium supplemented with (**A**) 2-phenylethyl-ITC (15 µM; PEITC) or (**B**) allyl-ITC (500 µM; AITC), before being transferred to new control medium and grown for another ten days. Two Way ANOVA and a post hoc Holm–Sidak test was used to determine significant differences at each time point. For the purpose of readability, only significant differences in root length between seedlings transferred to new control plates and those remaining on ITC-supplemented plates (marked by “a”) or those remaining on control plates (marked by “b”) are indicated (*n* = 48; * *p* < 0.001; error bars indicate SDs).

To assess if plants were able to recover from the ITC-triggered inhibition of root growth, seedlings were first grown on medium supplemented with either 500 µM allyl-ITC or 15 µM 2-phenylethyl-ITC, before being transferred to new control medium (Figure 6). After this transfer, roots grew more rapidly (6.80 ± 0.75 mm/day when transferred from allyl-ITC and 6.21 ± 1.39 mm/day when transferred from 2-phenylethyl-ITC) than those of seedlings still exposed to the ITC (4.50 ± 0.55 mm/day on allyl-ITC and 4.96 ± 0.99 mm/day on 2-phenylethyl-ITC), and reached growth rates similar to seedlings never exposed to ITC (i.e., transferred from control plates to new control plates; 7.53 ± 1.05 mm/day and 6.92 ± 1.32 mm/day). This was observed for both 2-phenylethyl-ITC (Figure 6A) and allyl-ITC (Figure 6B), showing that root growth can recover when the exogenously applied ITCs are removed.

## 3. Discussion

The aim of the present study was to assess the growth inhibiting effect of a range of ITCs on the model plant *A. thaliana* under standardized in vitro conditions. Three of the five ITCs used in this study are glucosinolate-derived compounds that occur naturally. Allyl-ITC is generated by the hydrolysis of the aliphatic glucosinolate sinigrin. Allyl-ITC was chosen as it can be generated by *A. thaliana* [47,48], although not by the Col-0 accession, and had been used in some earlier studies to assess the effects of ITCs on different plants. Benzyl-ITC and 2-phenylethyl-ITC are generated by the hydrolysis of the aromatic glucosinolates glucotropaeolin and gluconasturtiin, respectively. These glucosinolates are present at very low concentrations in some *A. thaliana* accessions only [49,50], but are readily produced in Brassicales species like *Tropaeolum majus* and *Nasturtium officinale*, respectively [51]. Glucosinolates that generate phenyl-ITC or 4-phenylbutyl-ITC probably do not occur naturally, despite early reports on their existence [3]. Using this series of five ITCs allowed us to assess if the structure and the length of their side chain has an impact on their effect on *A. thaliana* growth.

All five ITCs that were tested had a negative effect on plant growth, measured in our assays as a reduction in primary root length and total biomass. Our results confirmed earlier studies by showing that some glucosinolate-derived ITCs can be phytotoxic at high concentrations. By exposing in vitro-grown *A. thaliana* plants for 1 h to ITCs in the vapor phase, it was recently shown that allyl-ITC at the molar range led to leaf bleaching, reduced subsequent plant growth, and some lethality [22]. It had previously been shown that spraying vermiculite-grown *A. thaliana* plants with allyl-ITC at concentrations higher than 10 mM inhibited fresh weight, led to bleaching of rosette leaves and reduced chlorophyll content, while no effect was observed at a 1 mM concentration [15]. Our assays revealed that ITCs exert growth inhibitory effects at concentrations that are much lower than those previously used, and that root growth recovered from an exposure to ITCs. Concentrations of 100 µM allyl-ITC and phenyl-ITC, and 25 µM for three other aromatic ITCs (benzyl-ITC, 2-phenylethyl-ITC, and 4-phenylbutyl-ITC) showed a clear inhibitory effect on plant growth. Extrapolation of root growth inhibition data shown in Figure 5C also suggests that the aromatic ITCs benzyl-ITC and 2-phenylethyl-ITC will exert effects at doses lower than 25 µM. This was indeed confirmed by using 2-phenylethyl-ITC at 15 µM during the recovery assay (Figure 6).

Roots of *A. thaliana* Col-0 seedlings contain approximately 3.2 µmol of total aliphatic glucosinolates per g of dry weight, which would correspond to a concentration of approximately 320 µM [52]. The concentrations we tested could therefore be within the amount of ITCs generated by hydrolysis of root aliphatic glucosinolates upon damage, and even higher amounts of ITCs could be generated locally. Indeed, glucosinolates are not evenly distributed in *A. thaliana* tissues, and cells containing an estimated amount of glucosinolates that exceeds 100 mM have been identified [53,54,55,56]. The quantity and nature of glucosinolates present in plant tissue is affected by numerous parameters, including the plant species and cultivar, the organ and the growth stage of a plant, and environmental conditions [41,42,57,58]. *A. thaliana* accessions show large qualitative differences in glucosinolate profiles and up to 20-fold changes in the total amount of aliphatic glucosinolates in rosette leaves [49]. Between different organs of the accession Col-0, the total glucosinolate concentration varied nearly 100-fold [41]. The amount of ITCs that will be formed by hydrolysis of glucosinolates upon plant tissue disruption will therefore be influenced by these parameters, as well as the extent of the damage and the capacity of the plant to divert glucosinolate hydrolysis from ITC towards the formation of other products (e.g., nitriles and epithionitriles) [40]. Glucosinolates and their hydrolysis products have also been identified in exudates of healthy roots, but the reported amounts of ITCs were much lower than those having an inhibitory effect in our assays [59,60,61,62,63,64].

Although the five ITCs that we tested negatively affected plant growth, they exhibited differences in their potency to generate the observed growth inhibition, with the aromatic ITCs showing a greater effect than the aliphatic allyl-ITC. Based on the phenotype caused by the tested ITCs, they can be ranged as follows: 2-phenylethyl-ITC > benzyl-ITC > 4-phenylbutyl-ITC > phenyl-ITC > allyl-ITC (from the most potent to the least potent). Quantitative differences in the effects triggered by different ITCs have been reported earlier, although with variations between studies. Benzyl-ITC was more potent than three aliphatic ITCs (sulforaphane, butyl-ITC, isopropyl-ITC) in inducing electrolyte leakage, indicative of cell death, when syringe-infiltrated into soil-grown *A. thaliana* leaves [14]. Growth inhibition of *A. thaliana* seedlings was more severe when exposed for a short time to vapors of allyl-ITC than to those of 2-phenylethyl-ITC or benzyl-ITC [22], which is different from what we observed in the current study where seedlings were grown on medium supplemented with these ITCs. However, Hara et al. [15] also observed that 2-phenylethyl-ITC had stronger effects than allyl-ITC at equal doses when spraying vermiculite-grown *A. thaliana* plants with different ITCs, while the other tested compound (methyl-ITC) was much less potent. Effects of ITCs on the growth of plant species that do not produce glucosinolates have also been reported. Wheat coleoptile and root length was, for example, strongly inhibited when seeds were placed on filter paper wetted with 2-phenylethyl-ITC. Less inhibition was observed for allyl-ITC and benzyl-ITC, and phenyl-ITC had hardly any effect [65].

ITCs with different chemical structures and side chains differ in their physicochemical properties such as volatility, solubility, stability, reactivity, and ability to cross the cell membrane [66,67,68,69]. ITCs are reactive and unstable compounds, and allyl-ITC is known to decompose to allyl allyldithiocarbamate and to react with water to form allylamine, which then further reacts with residual allyl-ITC to form *N*,*N*′-diallylthiourea in aqueous solution, with parameters such as pH and temperature affecting allyl-ITC decomposition [69,70,71,72,73]. Under certain conditions, 2-phenylethyl-ITC and benzyl-ITC can also decompose into their respective amines phenethylamine and benzylamine [74,75]. ITCs are volatile compounds, but differ in their vapor pressure [76,77]. These differences might then be exacerbated by parameters of the experimental setup such as the mode of ITC application, the nature of the solvent, the properties of the plant growth substrate or the environmental conditions during testing. Also, the growth stage of the plant might affect susceptibility to the compound, the processes that are affected, and the responses that are triggered. In addition, some of the differences between the effects of different ITCs might be explained by the use of different plant species [78,79]. The above-mentioned and other studies illustrate the importance of experimental conditions when investigating the effect of ITCs on plants, hence our choice of testing the effects of ITCs on the model organism *A. thaliana* under a standard in vitro assay that is widely used for studying many other abiotic and chemical stressors. Nevertheless, the differences we observed with the tested ITCs as to their effects on growth parameters of *A. thaliana* seedlings must be interpreted taking into account differences in their physicochemical properties under our experimental conditions. Measurements were done 10 days after starting exposure to the indicated concentrations, and although the effects of ITCs can be observed at earlier time points (Figure 6), differential breakdown, reaction with other compounds in the medium and volatility of the chosen ITCs will have affected the exposure of seedlings during the treatment period.

The nature of the plant parameter(s) that are assessed might also affect the outcome of a study, as different ITCs might affect them differently [15,65]. In the current study, we measured the main root length and total biomass production and we observed major differences between ITCs but a good correlation between the effects of each ITC on these two parameters. The assessment of other parameters might have given a different ranking of the tested ITCs.

When comparing the four aromatic ITCs, we observed an increase in potency with the chain length from phenyl-ITC to benzyl-ITC and 2-phenylethyl-ITC. As 4-phenylbutyl-ITC was less potent than the latter two, our study revealed that there is an optimum of the side chain length as to the inhibitory effect of aromatic ITCs on *A. thaliana* growth. The side chain length of aromatic ITCs also had an impact on the germination and growth of wheat [65] and on the emergence of Palmer amaranth (*Amaranthus palmeri*) [78], but not on other plant species [13,78]. Interestingly, studies on the tumor-inhibiting effects of aromatic ITCs have also shown an increased effect with an increase in the side chain length up to 6-phenylhexyl-ITC (i.e., a C_6_ chain length) and a decline in the effect of ITCs with longer chains [80,81,82]. An increase in chain length might affect the delivery of ITCs to the target tissue, due to an increase in lipophilicity and a decrease in reactivity, and favor binding to their target enzyme [82].

The molecular mechanisms underlying the observed effects of ITCs on *A. thaliana* are not yet understood, but are currently investigated. It was recently shown that short-term exposure of *A. thaliana* to high allyl-ITC concentrations leads to disintegration of microtubules, inhibits actin-mediated intracellular transport, affects the cell cycle, and depletes the glutathione pool [17,21,22,23]. As mentioned before, ITC treatments lead to stomatal closure [19], and it had been previously proposed that ITCs might lead to the inhibition of inward K^+^ channels in guard cells to avoid water loss by stomatal closure in response to abiotic stress [83,84]. It has been shown that exogenous treatment with sinigrin, likely through the generation of allyl-ITC, affects aquaporins and regulates water transport under salt stress in *Brassica oleracea* [85]. That short-chain aliphatic glucosinolates may contribute to water saving under salt stress was also indicated by the double *A. thaliana* mutant *myb28myb29*, which lacks aliphatic glucosinolates and exhibited a greater reduction in the hydraulic conductivity under salt stress than wild type plants [86]. Whether any of these processes is involved in the longer-term growth inhibition observed here and whether differences between ITCs are due to a differential modulation of these processes needs further investigation. Here, we attempted to assess the involvement of the phytohormones auxin and ethylene in the ITC-triggered effects. Using the auxin marker lines DR5::GUS and DII-VENUS we did not find any indications that auxin levels changed after allyl-ITC treatment (Figure 4), and mutants affected in the auxin response did not exhibit a different response than wild type seedlings to allyl-ITC under the tested conditions. Similarly, ethylene insensitive mutants did not behave differently from wild type seedlings on ITC-supplemented medium. Hence, our results so far do not allow us to conclude if auxin and/or ethylene play a role in the allyl-ITC-triggered root growth inhibition. However, as previous transcriptional profiling has shown that the expression of many auxin responsive genes (*indole-3-acetic acid inducible* (*IAA*) and *small auxin upregulated* (*SAUR*)) were repressed and that many genes encoding ethylene response factors (ERFs) were affected by a short-term allyl-ITC treatment [20], it might be worth investigating these processes more thoroughly in the future. Interestingly, it was recently reported that treatment with the glucosinolate hydrolysis product indole-3-carbinol inhibited *A. thaliana* root elongation by interfering with auxin perception [87]. Considering that ITCs with different structures vary in their effect, as was observed in our study and has been reported in the literature, it can be expected that different glucosinolate hydrolysis products affect plant growth and development by different mechanisms. These remain, however, largely uncharacterized.

In the current study, we investigated the effect of different ITCs on the root growth and biomass of *A. thaliana* Col-0 under standard in vitro growth conditions that have been used for studying many other abiotic and chemical stress situations. This is part of our ongoing effort to characterize the morphogenic responses induced by ITCs and other glucosinolate hydrolysis products in more detail, to investigate further which molecular mechanisms underlie the observed effects (e.g., by using omics approaches, by assessing the response of mutant plants), and to test the antagonistic or synergistic effect with other environmental factors (e.g., light, temperature) or chemical compounds (e.g., hormones, abiotic stress inducers).

## 4. Materials and Methods

### 4.1. Isothiocyanates

The following ITCs were used during the course of this study: 2-propenyl-ITC (allyl-ITC; CAS 57-06-7; Sigma-Aldrich (St. Louis, MO, USA) 377430; 95%), phenyl-ITC (CAS 103-72-0; Sigma-Aldrich 139742; 98%), benzyl-ITC (CAS 622-78-6; Sigma-Aldrich 252492; 98%), 2-phenylethyl-ITC (phenethyl-ITC; CAS 2257-09-2; Sigma-Aldrich 253731; 99%), 4-phenylbutyl-ITC (CAS 61499-10-3; Maybridge, Altrincham, UK, TL00409EA; 95%).

### 4.2. Plant Material and Growth Conditions

*A. thaliana* accession Col-0 seeds were surface sterilized with chlorine gas for 4 h as described [88], and stratified for 3 days at 4 °C in water. Seeds were subsequently sown on square plates (120 × 120 × 17 mm, Greiner Bio One, Kremsmünster, Austria) containing 75 mL solid half strength Murashige & Skoog (MS) medium (2.15 g L^−1^ MS basal salt mixture (Sigma-Aldrich, M5524); 20 g L^−1^ sucrose; pH 5.7; 10 g L^−1^ 2/1 phyto agar (Duchefa Biochemie P1003, Haarlem, The Netherlands)/bacteriological agar (VWR 84609, Radnor, PA, USA)). Plates were sealed (3M Micropore Surgical Tape 1530-1) and placed in a vertical position into a controlled growth chamber (VB1514; Vötsch Industrietechnik, Balingen-Frommern, Germany) under a 16 h light (75 µmol m^−2^ s^−1^)/8 h dark regime at 22 °C/18 °C for 10 days. The light was supplied by a combination of Osram L 30W/830 (2400 lm) Lumilux Warm white and Osram L 30W/77 (1000 lm) Fluora fluorescent tubes in a 4:3 ratio.

### 4.3. Treatment with Isothiocyanates and Assessment of Growth Parameters

To assess the effect of ITCs on plant growth, seeds were in parallel sown on ½ MS medium as described above (control) and sown on ½ MS medium supplemented with one of the above-mentioned ITCs at the concentration(s) indicated in the text (ranging from 50 to 1000 µM for allyl-ITC and phenyl-ITC and from 1 to 300 µM for benzyl-ITC, 2-phenylethyl-ITC and 4-phenylbutyl-ITC). A 1 M solution of each ITC was freshly prepared in dimethyl sulfoxide (DMSO) and further diluted in DMSO, so that for each of the ITC concentrations, an equal volume of solution was added to the medium (0.1% *v*/*v* DMSO in final). The control plates consisted of medium supplemented with DMSO to 0.1%. The ITC and DMSO-mock solutions were added to the autoclaved medium just before pouring the plates. Four replicate plates were used for each treatment. Root growth was monitored by taking pictures of the plates after 10 days, and primary root length was measured using the ImageJ software (https://imagej.nih.gov/ij/) [89]. The root length of at least 12 seedlings from each of the four replicate plates (*n* = 4) was measured. The number of lateral roots was monitored under a Zeiss Axio Zoom.V16 stereo zoom microscope. The lateral root density was calculated as ratio between the number of lateral roots and the primary root length (*n* > 35). In addition, the average seedling biomass was assessed at day 10 by pooling 15 seedlings from each of the four plates (*n* = 4), and dividing that biomass by the number of seedlings. Significant differences in root growth, biomass and lateral root density between the control and treatment groups were tested with ANOVA (One- or Two-Way ANOVA, for further details see the figure legends) using the SigmaPlot (Systat Software; ver. 13.0, https://systatsoftware.com/) software.

### 4.4. Recovery of Root Growth from Isothiocyanate Exposure

To assess whether the root growth was able to recover from ITC-triggered inhibition, seedlings were grown as described above on medium supplemented with ITC (allyl-ITC at 500 µM or 2-phenylethyl-ITC at 15 µM) or control medium (4 replicate plates per treatment). On the fifth day, half of the seedlings from each plate were moved to new control plates. Root length was measured at the time of transfer (day 5) and at different time points after transfer (days 7, 10, 12 and 15).

### 4.5. Histochemical Analysis of GUS Activity

Detection of β-glucuronidase (GUS) activity was carried out as previously described [90], with minor modifications. Briefly, 10 day old DR5::GUS seedlings were incubated in 90% (*v*/*v*) pre-chilled acetone at −20 °C for 1 h. Seedlings were then washed twice for 5 min with 100 mM sodium phosphate buffer (pH 7.8) before GUS reaction buffer (100 mM sodium phosphate buffer, pH 7.8; 10 mM EDTA; 1 mM potassium ferrocyanide; 1 mM potassium ferricyanide; 1% Triton X-100) supplemented with 1 mM X-GlcA cyclohexylammonium salt (Duchefa Biochemie X1405) was added. After vacuum infiltration for 3 min, samples were incubated for 3 h in the dark at 37 °C. The enzymatic reaction was stopped by replacing the staining solution with a mixture of ethanol and acetic acid (3:1), and stored at room temperature overnight. Samples were kept in 70% (*v*/*v*) ethanol at 4 °C prior to further investigations. Seedlings were mounted onto glass slides and analyzed by using a Nikon Eclipse (E800) upright microscope equipped with DS-Ri1 camera head and Plan Fluor 40x/0.75 (working distance 0.72 mm) objective. Images were further processed by using the NIS-Elements (F 3.0), Adobe Illustrator CC and Adobe Photoshop CC software (http://www.adobe.com/products/catalog.html).

### 4.6. Confocal Laser Scanning Microscopy (CLSM) Analysis

Seedlings of the auxin marker line DII-VENUS and the control line mDII-VENUS, grown on allyl-ITC-supplemented medium for 10 days, were rinsed in sterile water and mounted onto glass slides using water as a medium. Samples were investigated under a Leica (DMI 6000 CS Bino inverted microscope with Adaptive Focus Control) True Confocal Scanner (TCS) SP8 instrument equipped with single molecule detection (SMD) and multiphoton laser (MP) platforms. HCX IRAPO L 25x/0.95 water immersion objective (working distance 2.4 mm) was used with the pinhole size set to 1 airy unit (AU). The yellow fluorescent protein (YFP) variant VENUS was excited by a white light laser (WLL) at 514 nm and detected at 524–540 nm using sensitive hybrid detector (HyD). Images were processed by Leica Application Suite X (LAS X 2.0), Adobe Photoshop CC and Adobe Illustrator CC software.

## Figures and Tables

**Table 1:** List of auxin and ethylene mutants used to investigate the role of these hormones in allyl-ITC-triggered growth inhibition. Root length of seedlings grown on ½ Murashige & Skoog (MS) medium or ½ MS medium supplemented with allyl-ITC was measured on day 10. Allyl-ITC concentrations tested were 50, 100, 300, and 500 µM, except for *ein2-1* and *etr1-3*, where 500 µM allyl-ITC was used.

Mutant Name	Gene Name (ID)	Description
*eir1-1 (pin2-1)*	*EIR1/PIN2* (At5g57090)	auxin efflux carrier
*pin3-4*	*PIN3* (At1g70940)	regulator of auxin efflux and transport
*pin4-3*	*PIN4* (At2g01420)	putative auxin efflux carrier
*tir1-1*	*TIR1* (At3g62980)	auxin receptor/F-box protein
*axr2-1*	*AXR2/IAA7* (At3g23050)	member of Aux/IAA protein family
*axr3-1*	*AXR3/IAA17* (At1g04250)	member of Aux/IAA protein family
*axr4-1*	*AXR4* (At1g54990)	involved in polar auxin transport
*axr6-1*	*AXR6* (At4g02570)	CUL1 subunit of SCF complex
*ein2-1*	*EIN2* (At5g03280)	involved in ethylene signal transduction
*etr1-3*	*ETR1/EIN1* (At1g66340)	ethylene binding receptor

## Supplementary Materials

Supplementary materials can be found at www.mdpi.com/1422-0067/18/11/2372/s1.

## References

[1] Bones A.M., Rossiter J.T. (1996). The myrosinase-glucosinolate system, its organisation and biochemistry. Physiol. Plant..

[2] Kissen R., Rossiter J.T., Bones A.M. (2009). The “mustard oil bomb”: Not so easy to assemble?! Localization, expression and distribution of the components of the myrosinase enzyme system. Phytochem. Rev..

[3] Agerbirk N., Olsen C.E. (2012). Glucosinolate structures in evolution. Phytochemistry.

[4] Bones A.M., Rossiter J.T. (2006). The enzymic and chemically induced decomposition of glucosinolates. Phytochemistry.

[5] Kong X.Y., Kissen R., Bones A.M. (2012). Characterization of recombinant nitrile-specifier proteins (NSPs) of *Arabidopsis thaliana*: Dependency on Fe(II) ions and the effect of glucosinolate substrate and reaction conditions. Phytochemistry.

[6] Zabala M.D., Grant M., Bones A.M., Bennett R., Lim Y.S., Kissen R., Rossiter J.T. (2005). Characterisation of recombinant epithiospecifier protein and its over-expression in *Arabidopsis thaliana*. Phytochemistry.

[7] Dinkova-Kostova A.T., Kostov R.V. (2012). Glucosinolates and isothiocyanates in health and disease. Trends Mol. Med..

[8] Traka M., Mithen R. (2009). Glucosinolates, isothiocyanates and human health. Phytochem. Rev..

[9] Hopkins R.J., van Dam N.M., van Loon J.J.A. (2009). Role of glucosinolates in insect-plant relationships and multitrophic interactions. Annu. Rev. Entomol..

[10] Piasecka A., Jedrzejczak-Rey N., Bednarek P. (2015). Secondary metabolites in plant innate immunity: Conserved function of divergent chemicals. New Phytol..

[11] Gimsing A.L., Kirkegaard J.A. (2009). Glucosinolates and biofumigation: Fate of glucosinolates and their hydrolysis products in soil. Phytochem. Rev..

[12] Norsworthy J.K., Meehan J.T. (2005). Herbicidal activity of eight isothiocyanates on Texas panicum (*Panicum texanum*), large crabgrass (*Digitaria sanguinalis*), and sicklepod (*Senna obtusifolia*). Weed Sci..

[13] Vaughn S., Boydston R. (1997). Volatile allelochemicals released by crucifer green manures. J. Chem. Ecol..

[14] Andersson M.X., Nilsson A.K., Johansson O.N., Boztas G., Adolfsson L.E., Pinosa F., Petit C.G., Aronsson H., Mackey D., Tor M. (2015). Involvement of the electrophilic isothiocyanate sulforaphane in Arabidopsis local defense responses. Plant Physiol..

[15] Hara M., Yatsuzuka Y., Tabata K., Kuboi T. (2010). Exogenously applied isothiocyanates enhance glutathione S-transferase expression in Arabidopsis but act as herbicides at higher concentrations. J. Plant Physiol..

[16] Rivera-Vega L.J., Krosse S., de Graaf R.M., Garvi J., Garvi-Bode R.D., van Dam N.M. (2015). Allelopathic effects of glucosinolate breakdown products in Hanza [*Boscia senegalensis* (Pers.) Lam.] processing waste water. Front. Plant Sci..

[17] Øverby A., Stokland R.A., Asberg S.E., Sporsheim B., Bones A.M. (2015). Allyl isothiocyanate depletes glutathione and upregulates expression of glutathione S-transferases in *Arabidopsis thaliana*. Front. Plant Sci..

[18] Hossain M.S., Ye W., Hossain M.A., Okuma E., Uraji M., Nakamura Y., Mori I.C., Murata Y. (2013). Glucosinolate degradation products, isothiocyanates, nitriles, and thiocyanates, induce stomatal closure accompanied by peroxidase-mediated reactive oxygen species production in *Arabidopsis thaliana*. Biosci. Biotechnol. Biochem..

[19] Khokon M.A., Jahan M.S., Rahman T., Hossain M.A., Muroyama D., Minami I., Munemasa S., Mori I.C., Nakamura Y., Murata Y. (2011). Allyl isothiocyanate (AITC) induces stomatal closure in Arabidopsis. Plant Cell Environ..

[20] Kissen R., Overby A., Winge P., Bones A.M. (2016). Allyl-isothiocyanate treatment induces a complex transcriptional reprogramming including heat stress, oxidative stress and plant defence responses in *Arabidopsis thaliana*. BMC Genom..

[21] Sporsheim B., Øverby A., Bones A.M. (2015). Allyl isothiocyanate inhibits actin-dependent intracellular transport in *Arabidopsis thaliana*. Int. J. Mol. Sci..

[22] Øverby A., Baevre M.S., Thangstad O.P., Bones A.M. (2015). Disintegration of microtubules in *Arabidopsis thaliana* and bladder cancer cells by isothiocyanates. Front. Plant Sci..

[23] Åsberg S.E., Bones A.M., Øverby A. (2015). Allyl isothiocyanate affects the cell cycle of *Arabidopsis thaliana*. Front. Plant Sci..

[24] Overvoorde P., Fukaki H., Beeckman T. (2010). Auxin control of root development. Cold Spring Harb. Perspect. Biol..

[25] Petricka J.J., Winter C.M., Benfey P.N. (2012). Control of Arabidopsis root development. Annu. Rev. Plant Biol..

[26] Kazan K. (2013). Auxin and the integration of environmental signals into plant root development. Ann. Bot..

[27] Perrot-Rechenmann C. (2010). Cellular responses to auxin: Division versus expansion. Cold Spring Harb. Perspect. Biol..

[28] Takatsuka H., Umeda M. (2014). Hormonal control of cell division and elongation along differentiation trajectories in roots. J. Exp. Bot..

[29] Fukaki H., Tasaka M. (2009). Hormone interactions during lateral root formation. Plant Mol. Biol..

[30] Marchant A., Bhalerao R., Casimiro I., Eklof J., Casero P.J., Bennett M., Sandberg G. (2002). AUX1 promotes lateral root formation by facilitating indole-3-acetic acid distribution between sink and source tissues in the Arabidopsis seedling. Plant Cell.

[31] Swarup K., Benkova E., Swarup R., Casimiro I., Peret B., Yang Y., Parry G., Nielsen E., de Smet I., Vanneste S. (2008). The auxin influx carrier LAX3 promotes lateral root emergence. Nat. Cell Biol..

[32] Benkova E., Michniewicz M., Sauer M., Teichmann T., Seifertova D., Jurgens G., Friml J. (2003). Local, efflux-dependent auxin gradients as a common module for plant organ formation. Cell.

[33] Blilou I., Xu J., Wildwater M., Willemsen V., Paponov I., Friml J., Heidstra R., Aida M., Palme K., Scheres B. (2005). The PIN auxin efflux facilitator network controls growth and patterning in Arabidopsis roots. Nature.

[34] Swarup R., Perry P., Hagenbeek D., van der Straeten D., Beemster G.T., Sandberg G., Bhalerao R., Ljung K., Bennett M.J. (2007). Ethylene upregulates auxin biosynthesis in Arabidopsis seedlings to enhance inhibition of root cell elongation. Plant Cell.

[35] Ruzicka K., Ljung K., Vanneste S., Podhorska R., Beeckman T., Friml J., Benkova E. (2007). Ethylene regulates root growth through effects on auxin biosynthesis and transport-dependent auxin distribution. Plant Cell.

[36] Ortega-Martinez O., Pernas M., Carol R.J., Dolan L. (2007). Ethylene modulates stem cell division in the *Arabidopsis thaliana* root. Science.

[37] Muday G.K., Rahman A., Binder B.M. (2012). Auxin and ethylene: Collaborators or competitors?. Trends Plant Sci..

[38] Lewis D.R., Negi S., Sukumar P., Muday G.K. (2011). Ethylene inhibits lateral root development, increases IAA transport and expression of PIN3 and PIN7 auxin efflux carriers. Development.

[39] Negi S., Ivanchenko M.G., Muday G.K. (2008). Ethylene regulates lateral root formation and auxin transport in *Arabidopsis thaliana*. Plant J..

[40] Wittstock U., Burow M. (2010). Glucosinolate breakdown in Arabidopsis: Mechanism, regulation and biological significance. Arabidopsis Book.

[41] Brown P.D., Tokuhisa J.G., Reichelt M., Gershenzon J. (2003). Variation of glucosinolate accumulation among different organs and developmental stages of *Arabidopsis thaliana*. Phytochemistry.

[42] Clossais-Besnard N., Larher F. (1991). Physiological role of glucosinolates in *Brassica napus*. Concentration and distribution pattern of glucosinolates among plant organs during a complete life cycle. J. Sci. Food Agric..

[43] James D.C., Rossiter J.T. (1991). Development and characteristics of myrosinase in *Brassica napus* during early seedling growth. Physiol. Plant..

[44] Stepanova A.N., Yun J., Likhacheva A.V., Alonso J.M. (2007). Multilevel interactions between ethylene and auxin in Arabidopsis roots. Plant Cell.

[45] Ulmasov T., Murfett J., Hagen G., Guilfoyle T.J. (1997). Aux/IAA proteins repress expression of reporter genes containing natural and highly active synthetic auxin response elements. Plant Cell.

[46] Brunoud G., Wells D.M., Oliva M., Larrieu A., Mirabet V., Burrow A.H., Beeckman T., Kepinski S., Traas J., Bennett M.J. (2012). A novel sensor to map auxin response and distribution at high spatio-temporal resolution. Nature.

[47] Kissen R., Pope T.W., Grant M., Pickett J.A., Rossiter J.T., Powell G. (2009). Modifying the alkylglucosinolate profile in *Arabidopsis thaliana* alters the tritrophic interaction with the herbivore Brevicoryne brassicae and parasitoid Diaeretiella rapae. J. Chem. Ecol..

[48] Lambrix V., Reichelt M., Mitchell-Olds T., Kliebenstein D.J., Gershenzon J. (2001). The Arabidopsis epithiospecifier protein promotes the hydrolysis of glucosinolates to nitriles and influences Trichoplusia ni herbivory. Plant Cell.

[49] Kliebenstein D.J., Kroymann J., Brown P., Figuth A., Pedersen D., Gershenzon J., Mitchell-Olds T. (2001). Genetic control of natural variation in Arabidopsis glucosinolate accumulation. Plant Physiol..

[50] Reichelt M., Brown P.D., Schneider B., Oldham N.J., Stauber E., Tokuhisa J., Kliebenstein D.J., Mitchell-Olds T., Gershenzon J. (2002). Benzoic acid glucosinolate esters and other glucosinolates from *Arabidopsis thaliana*. Phytochemistry.

[51] Fahey J.W., Zalcmann A.T., Talalay P. (2001). The chemical diversity and distribution of glucosinolates and isothiocyanates among plants. Phytochemistry.

[52] Petersen B.L., Chen S., Hansen C.H., Olsen C.E., Halkier B.A. (2002). Composition and content of glucosinolates in developing *Arabidopsis thaliana*. Planta.

[53] Sarsby J., Towers M.W., Stain C., Cramer R., Koroleva O.A. (2012). Mass spectrometry imaging of glucosinolates in Arabidopsis flowers and siliques. Phytochemistry.

[54] Shroff R., Vergara F., Muck A., Svatos A., Gershenzon J. (2008). Nonuniform distribution of glucosinolates in *Arabidopsis thaliana* leaves has important consequences for plant defense. Proc. Natl. Acad. Sci. USA.

[55] Koroleva O.A., Davies A., Deeken R., Thorpe M.R., Tomos A.D., Hedrich R. (2000). Identification of a new glucosinolate-rich cell type in Arabidopsis flower stalk. Plant Physiol..

[56] Koroleva O.A., Gibson T.M., Cramer R., Stain C. (2010). Glucosinolate-accumulating S-cells in Arabidopsis leaves and flower stalks undergo programmed cell death at early stages of differentiation. Plant J..

[57] Hanschen F.S., Schreiner M. (2017). Isothiocyanates, nitriles, and epithionitriles from glucosinolates are affected by genotype and developmental stage in Brassica oleracea varieties. Front. Plant Sci..

[58] Yan X.F., Chen S.X. (2007). Regulation of plant glucosinolate metabolism. Planta.

[59] Rumberger A., Marschner P. (2003). 2-Phenylethylisothiocyanate concentration and microbial community composition in the rhizosphere of canola. Soil Biol. Biochem..

[60] Schreiner M., Krumbein A., Knorr D., Smetanska I. (2011). Enhanced glucosinolates in root exudates of *Brassica rapa* ssp. rapa mediated by salicylic acid and methyl jasmonate. J. Agric. Food Chem..

[61] Strehmel N., Böttcher C., Schmidt S., Scheel D. (2014). Profiling of secondary metabolites in root exudates of *Arabidopsis thaliana*. Phytochemistry.

[62] Auger B., Pouvreau J.B., Pouponneau K., Yoneyama K., Montiel G., Le Bizec B., Delavault P., Delourme R., Simier P. (2012). Germination stimulants of *Phelipanche ramosa* in the rhizosphere of *Brassica napus* are derived from the glucosinolate pathway. Mol. Plant Microbe Interact..

[63] Mönchgesang S., Strehmel N., Schmidt S., Westphal L., Taruttis F., Müller E., Herklotz S., Neumann S., Scheel D. (2016). Natural variation of root exudates in *Arabidopsis thaliana*-linking metabolomic and genomic data. Sci. Rep..

[64] Xu D., Hanschen F.S., Witzel K., Nintemann S.J., Nour-Eldin H.H., Schreiner M., Halkier B.A. (2017). Rhizosecretion of stele-synthesized glucosinolates and their catabolites requires GTR-mediated import in Arabidopsis. J. Exp. Bot..

[65] Bialy Z., Oleszek W., Lewis J., Fenwick G.R. (1990). Allelopathic potential of glucosinolates (mustard oil glycosides) and their degradation products against wheat. Plant Soil.

[66] Hanschen F.S., Lamy E., Schreiner M., Rohn S. (2014). Reactivity and stability of glucosinolates and their breakdown products in foods. Angew. Chem. Int. Ed..

[67] Jiao D., Eklind K.I., Choi C.I., Desai D.H., Amin S.G., Chung F.L. (1994). Structure-activity relationships of isothiocyanates as mechanism-based inhibitors of 4-(methylnitrosamino)-1-(3-pyridyl)-1-butanone-induced lung tumorigenesis in A/J mice. Cancer Res..

[68] Luang-In V., Rossiter J.T. (2015). Stability studies of isothiocyanates and nitriles in aqueous media. Songklanakarin J. Sci. Technol..

[69] Ohta Y., Takatani K., Kawakishi S. (1995). Decomposition rate of allyl isothiocyanate in aqueous solution. Biosci. Biotechnol. Biochem..

[70] Kawakishi S., Namiki M. (1969). Decomposition of allyl isothiocyanate in aqueous solution. Agric. Biol. Chem..

[71] Pecháček R., Velíšek J., Hrabcová H. (1997). Decomposition products of allyl isothiocyanate in aqueous solutions. J. Agric. Food Chem..

[72] Tsao R., Yu Q., Friesen I., Potter J., Chiba M. (2000). Factors affecting the dissolution and degradation of oriental mustard-derived sinigrin and allyl isothiocyanate in aqueous media. J. Agric. Food Chem..

[73] Hanschen F.S., Bauer A., Mewis I., Keil C., Schreiner M., Rohn S., Kroh L.W. (2012). Thermally induced degradation of aliphatic glucosinolates: Identification of intermediary breakdown products and proposed degradation pathways. J. Agric. Food Chem..

[74] De Nicola G.R., Montaut S., Rollin P., Nyegue M., Menut C., Iori R., Tatibouet A. (2013). Stability of benzylic-type isothiocyanates in hydrodistillation-mimicking conditions. J. Agric. Food Chem..

[75] Song L., Iori R., Thornalley P.J. (2006). Purification of major glucosinolates from Brassicaceae seeds and preparation of isothiocyanate and amine metabolites. J. Sci. Food Agric..

[76] Rohloff J., Bones A.M. (2005). Volatile profiling of *Arabidopsis thaliana* - putative olfactory compounds in plant communication. Phytochemistry.

[77] Rumble J. (2017). CRC Handbook of Chemistry and Physics.

[78] Norsworthy J.K., Meehan J.T. (2005). Use of isothiocyanates for suppression of Palmer amaranth (*Amaranthus palmeri* ), pitted morningglory (*Ipomoea lacunosa*), and yellow nutsedge (*Cyperus esculentus*). Weed Sci..

[79] Wu H., Feng J.T., Lin K.C., Zhang X. (2012). Synthesis and herbicidal activity of substituted pyrazole isothiocyanates. Molecules.

[80] Conaway C.C., Jiao D., Chung F.-L. (1996). Inhibition of rat liver cytochrome P450 isozymes by isothiocyanates and their conjugates: A structure-activity relationship study. Carcinogenesis.

[81] Morse M.A., Eklind K.I., Amin S.G., Hecht S.S., Chung F.L. (1989). Effects of alkyl chain length on the inhibition of NNK-induced lung neoplasia in A/J mice by arylalkyl isothiocyanates. Carcinogenesis.

[82] Morse M.A., Eklind K.I., Hecht S.S., Jordan K.G., Choi C.I., Desai D.H., Amin S.G., Chung F.L. (1991). Structure-activity relationships for inhibition of 4-(methylnitrosamino)-1-(3-pyridyl)-1-butanone lung tumorigenesis by arylalkyl isothiocyanates in A/J mice. Cancer Res..

[83] Zhao Z., Zhang W., Stanley B.A., Assmann S.M. (2008). Functional proteomics of *Arabidopsis thaliana* guard cells uncovers new stomatal signaling pathways. Plant Cell.

[84] Del Carmen Martínez-Ballesta M., Moreno D.A., Carvajal M. (2013). The physiological importance of glucosinolates on plant response to abiotic stress in Brassica. Int. J. Mol. Sci..

[85] Martinez-Ballesta Mdel C., Muries B., Moreno D.A., Dominguez-Perles R., Garcia-Viguera C., Carvajal M. (2014). Involvement of a glucosinolate (sinigrin) in the regulation of water transport in *Brassica oleracea* grown under salt stress. Physiol. Plant..

[86] Martinez-Ballesta M., Moreno-Fernandez D.A., Castejon D., Ochando C., Morandini P.A., Carvajal M. (2015). The impact of the absence of aliphatic glucosinolates on water transport under salt stress in *Arabidopsis thaliana*. Front. Plant Sci..

[87] Katz E., Nisani S., Yadav B.S., Woldemariam M.G., Shai B., Obolski U., Ehrlich M., Shani E., Jander G., Chamovitz D.A. (2015). The glucosinolate breakdown product indole-3-carbinol acts as an auxin antagonist in roots of *Arabidopsis thaliana*. Plant J..

[88] Clough S.J., Bent A.F. (1998). Floral dip: A simplified method for Agrobacterium-mediated transformation of *Arabidopsis thaliana*. Plant J..

[89] Schneider C.A., Rasband W.S., Eliceiri K.W. (2012). NIH Image to ImageJ: 25 years of image analysis. Nat. Methods.

[90] Scarpella E., Francis P., Berleth T. (2004). Stage-specific markers define early steps of procambium development in Arabidopsis leaves and correlate termination of vein formation with mesophyll differentiation. Development.

