# Supplementary material for: Glucosinolate-Derived Isothiocyanates Inhibit Arabidopsis Growth and the Potency Depends on Their Side Chain Structure
Source: Int J Mol Sci. 2017 Nov 8;18(11):2372. doi: 10.3390/ijms18112372 (PMC5713341; doi:10.3390/ijms18112372)
Supplement: Supplementary file 1 [file ijms-18-02372-s001.pdf]

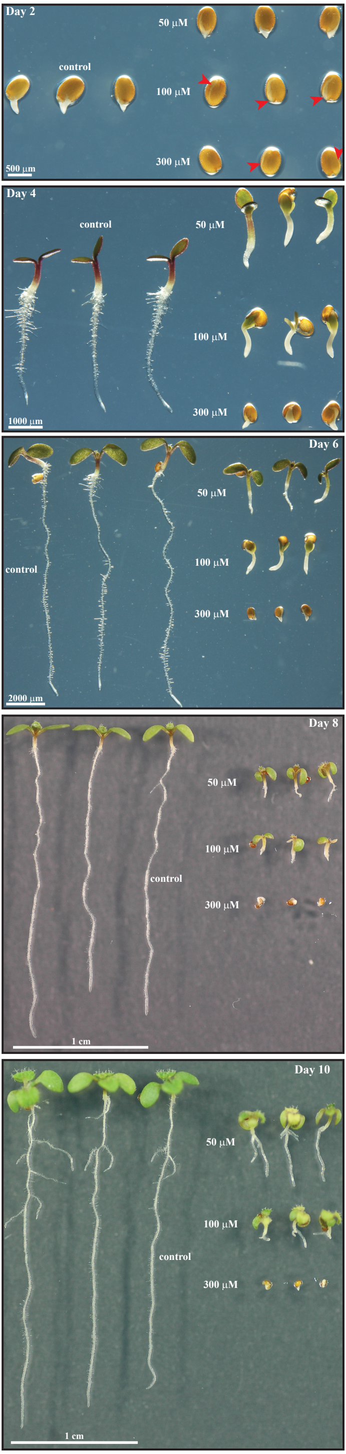

Figure S1. Time-course study on Col-0 WT plants following PEITC treatment. Representative individuals originated from plates supplemented with different concentrations of the phytochemical are shown at different time points (top corners), reflecting the actual developmental stage under the treatment. Red arrowheads indicate where the germination is initiated. Scale bars as displayed.
